# Wyllieite-type Ag_1.09_Mn_3.46_(AsO_4_)_3_

**DOI:** 10.1107/S1600536812018843

**Published:** 2012-05-02

**Authors:** Wafa Frigui, Mohamed Faouzi Zid, Ahmed Driss

**Affiliations:** a Laboratoire de Matériaux et Cristallochimie, Faculté des Sciences de Tunis, Université de Tunis ElManar, 2092 Manar II Tunis, Tunisia

## Abstract

Single crystals of wyllieite-type silver(I) manganese(II) tris­orthoarsenate(V), Ag_1.09_Mn_3.46_(AsO_4_)_3_, were grown by a solid-state reaction. The three-dimensional framework is made up from four Mn^2+^/Mn^3+^ cations surrounded octa­hedrally by O atoms. The MnO_6_ octa­hedra are linked through edge- and corner-sharing. Three independent AsO_4_ tetra­hedra are linked to the framework through common corners, delimiting channels along [100] in which two partly occupied Ag^+^ sites reside, one on an inversion centre and with an occupancy of 0.631 (4), the other on a general site and with an occupancy of 0.774 (3), both within distorted tetra­hedral environments. One of the Mn sites is also located on an inversion centre and is partly occupied, with an occupancy of 0.916 (5). Related compounds with alluaudite-type or rosemaryite-type structures are compared and discussed.

## Related literature

For background to framework structures with tetra­hedral and octa­hedral building units, see: Leclaire *et al.* (2002 ▶); Lii *et al.* (1990 ▶); Haddad *et al.* (1992 ▶); Hajji & Zid (2006 ▶); Borel *et al.* (1997 ▶); Masquelier *et al.* (1995 ▶). For details of structural relationships with other compounds, see: Warner *et al.* (1993 ▶); Korzenski *et al.* (1998 ▶); Chouaibi *et al.* (2001 ▶); Pertlik (1987 ▶); Antenucci *et al.* (1995 ▶); Zid *et al.* (2005 ▶); Ayed *et al.* (2004 ▶); MacKay *et al.* (1996 ▶); Alvarez-Vega *et al.* (2006 ▶); Frigui *et al.* (2010 ▶, 2011*a* ▶); Hatert (2006 ▶); Moore & Ito (1973 ▶, 1979 ▶); Yakubovich *et al.* (2005 ▶); Fransolet (1995 ▶); Moore & Molin-Case (1974 ▶). For preparative details, see: Frigui *et al.* (2010 ▶, 2011**a* ▶,b* ▶). For the bond-valence method, see: Brown & Altermatt (1985 ▶).

## Experimental

### 

#### Crystal data

Ag_1.09_Mn_3.46_(AsO_4_)_3_
*M* *_r_* = 724.02
Monoclinic, 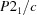

*a* = 6.7470 (7) Å
*b* = 12.9820 (9) Å
*c* = 11.2970 (8) Å
β = 98.85 (3)°
*V* = 977.72 (17) Å^3^
*Z* = 4
Mo *K*α radiation
μ = 16.64 mm^−1^
*T* = 298 K
0.25 × 0.15 × 0.10 mm

#### Data collection

Enraf–Nonius CAD-4 diffractometer
Absorption correction: ψ scan (North *et al.*, 1968 ▶) *T*_min_ = 0.071, *T*_max_ = 0.210
2522 measured reflections
2138 independent reflections
1704 reflections with *I* > 2σ(*I*)
*R*_int_ = 0.038
2 standard reflections every 120 min intensity decay: 1.1%

#### Refinement

*R*[*F* ^2^ > 2σ(*F* ^2^)] = 0.032
*wR*(*F* ^2^) = 0.098
*S* = 1.08
2138 reflections
188 parameters
1 restraint
Δρ_max_ = 1.08 e Å^−3^
Δρ_min_ = −1.37 e Å^−3^

### 

Data collection: *CAD-4 EXPRESS* (Duisenberg, 1992 ▶; Macíček & Yordanov, 1992 ▶); cell refinement: *CAD-4 EXPRESS*; data reduction: *XCAD4* (Harms & Wocadlo, 1995 ▶); program(s) used to solve structure: *SHELXS97* (Sheldrick, 2008 ▶); program(s) used to refine structure: *SHELXL97* (Sheldrick, 2008 ▶); molecular graphics: *DIAMOND* (Brandenburg, 1998 ▶); software used to prepare material for publication: *WinGX* (Farrugia, 1999 ▶).

## Supplementary Material

Crystal structure: contains datablock(s) I, global. DOI: 10.1107/S1600536812018843/wm2611sup1.cif

Structure factors: contains datablock(s) I. DOI: 10.1107/S1600536812018843/wm2611Isup2.hkl

Additional supplementary materials:  crystallographic information; 3D view; checkCIF report

## Figures and Tables

**Fig. 1.**
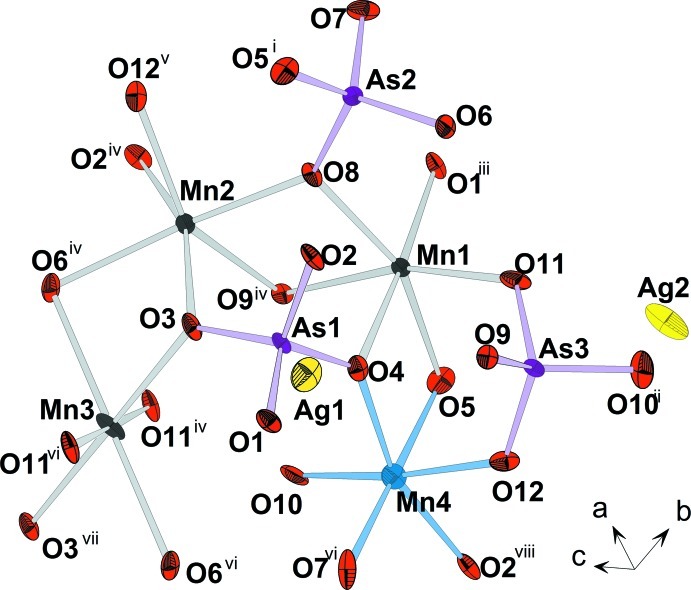
Unité asymétrique dans Ag1.09Mn3.46(AsO4)3. Les éllipsoïdes ont été définis avec 50% de probabilité. [Code de symétrie]: (i) 1 + x,y,z; (ii) x,1/2 - y,z- 1/2; (iii) 1 - x,1/2 + y,1/2 - z; (iv) x,1/2 - y,1/2 + z; (v) 1 + x,1/2 - y,1/2 + z; (vi) 1 - x,-1/2 + y,1/2 - z; (vii) 1 - x,-y,1 - z; (viii) -1 + x,y,z.

**Fig. 2.**
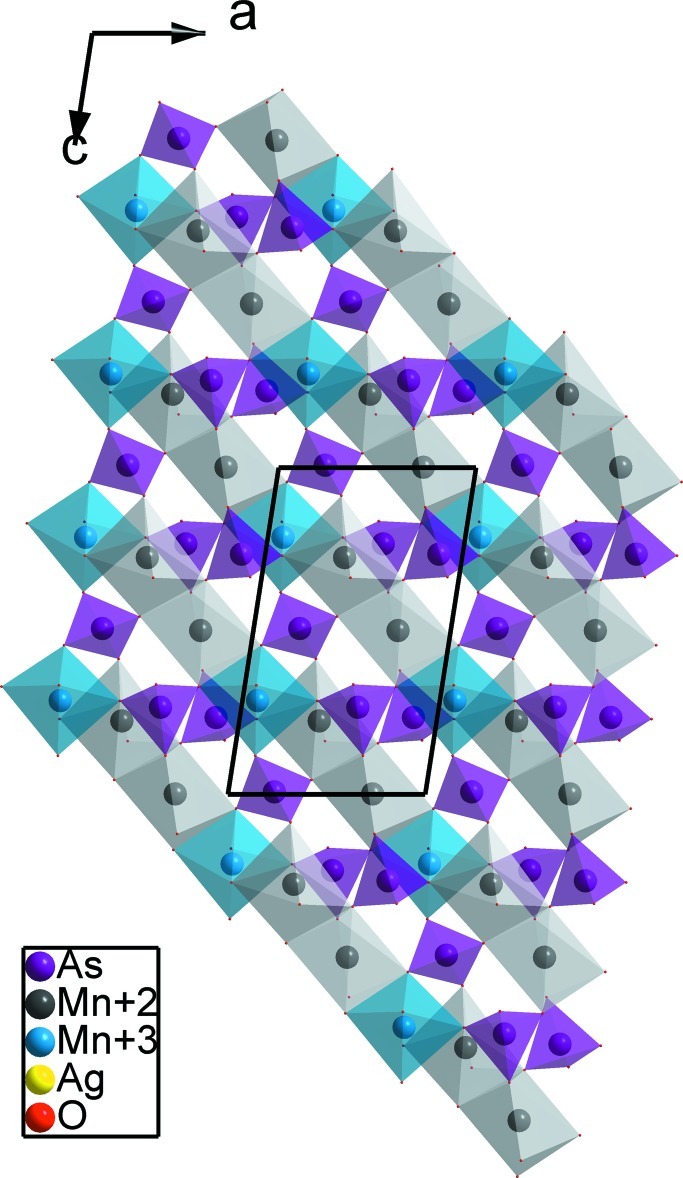
Représentation des couches infinies parallèles au plan (010)

**Fig. 3.**
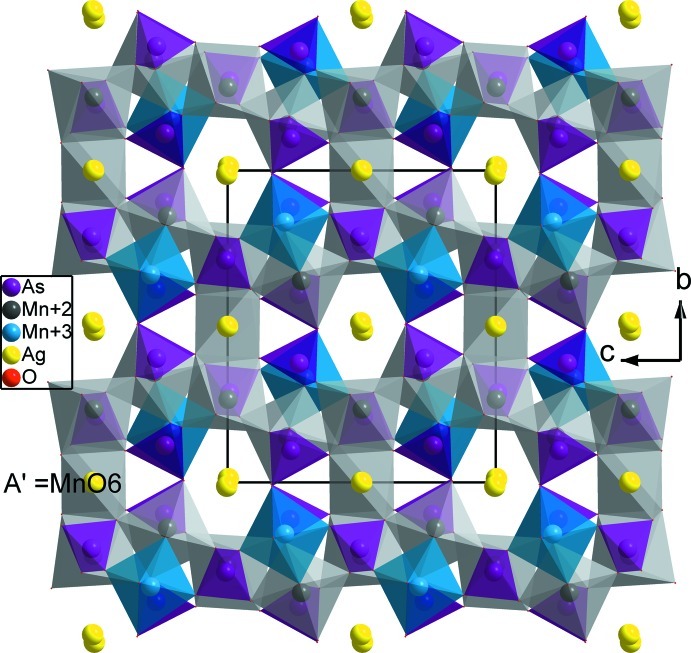
Projection de la structure de Ag1.09Mn3.46(AsO4)3 selon a mettant en evidence les cannaux où logent les cations.

**Fig. 4.**
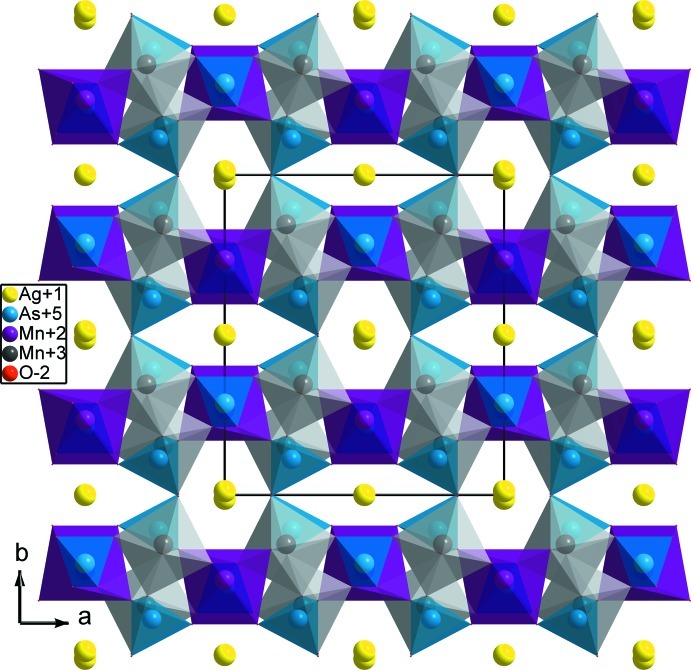
Projection de la structure, selon c, de Ag1.49Mn3(AsO4)3

## supplementary crystallographic information

### Comment

La recherche de nouveaux matériaux inorganiques à charpentes ouvertes formées d'octaèdres et de tétraèdres est actuellement un domaine d'intense activité (Borel *et al.*, 1997; Leclaire *et al.*, 2002; Lii *et al.*, 1990; Haddad *et al.*, 1992; Masquelier *et al.*, 1995; Hajji & Zid, 2006).

C'est dans ce cadre, que nous avons exploré les systèmes *A*–Mn–As–O (*A* = ion monovalent) qui demeurent relativement peu connus (Frigui *et al.*, 2010, 2011**a*,b*). Une nouvelle phase de formulation Ag_1.09_Mn_3.46_(AsO_4_)_3_ a été synthétisée par réaction à l'état solide. Un examen bibliographique montre que la structure est de type wyllieite (Moore & Ito, 1973, 1979; Fransolet, 1995; Yakubovich *et al.*, 2005;). Elle peut être décrite à partir de l'unité asymétrique 'Ag_2_Mn_4_As_3_O_12_' (Fig. 1). Elle est construite à partir de trois octaèdres MnO_6_ qui se lient par partage d'arêtes pour former le groupement Mn_3_O_14_. Ce dernier est lié à l'octaèdre Mn3O_6_ et trois tétraèdres AsO_4_ au moyen de sommets. Dans la charpente anionique les octaèdres MnO_6_ forment, par partage d'arêtes, des chaînes infinies (Mn1–Mn2–Mn4)_n_ disposées selon la direction [101]. Ces dernières se lient au moyen des tétraèdres AsO_4_ pour former des couches infinies parallèles au plan (010) (Fig. 2). Elles sont connectées d'une part, par des ponts mixtes Mn–O–As et d'autre part par partage d'arêtes avec l'octaèdre MnO_6_. Il en résulte une charpente tridimensionnelle, possédant des canaux parallèles à l'axe *a* où logent les cations Ag^+^ (Fig. 3). *L*'examen des facteurs géométriques dans la structure révèle qu'ils sont conformes avec ceux rencontrés dans la littérature (Ayed *et al.*, 2004; MacKay *et al.*, 1996; Alvarez-Vega *et al.*, 2006; Frigui *et al.*, 2010, 2011*a*). De plus, le calcul des différentes valences de liaison (BVS), utilisant la formule empirique de Brown (Brown & Altermatt, 1985), conduit aux valeurs des charges des ions suivants: Ag1(0,91), Ag2(1,06), As1(5,12), As2(5,04), As3(5,11), Mn1(2,11), Mn2(1,85), Mn3(1,69) e t Mn4 (2,63). La recherche de structures présentant des aspects communs avec celle de Ag_1.09_Mn_3.46_(AsO_4_)_3_, nous a conduit à la famille des alluaudites (Fig. 4) (Ayed *et al.*, 2004; Warner *et al.*, 1993; Korzenski *et al.*, 1998; Chouaibi *et al.*, 2001; Pertlik, 1987; Antenucci *et al.*, 1995; Zid *et al.*, 2005; Hatert, 2006). Les formules de ces deux minéraux, suggérées par Moore (Moore & Ito, 1979) sont *X*1_4_*X*2_4_*M*1_4_*M*2_8_(PO_4_)_12_ pour les alluaudites, et *Xa*_2_*X*1*b*_2_*X*2_4_*M*1_4_*X*2*a*_4_*M*2*b*_4_(PO_4_)_12_ pour les wyllieites, où *X* et *M* indiquent les positions des cations selon l'ordre de la taille des ions suivant: *M*2 < *M*1 < *X*1 < *X*2. Les formes alluaudite, rosemaryite et wyllieite cristallisent dans le même système monoclinique et présentent des paramètres de mailles similaires, cependant une différence cristallographique nette est observée dans la nature du type de réseaux de Bravais. En effet, le groupe d'espace choisi pour les structures type wyllieite ou bien rosemaryite est *P*2_1_/*c* (ou *P*2_1_/*n*) et celui adopté pour la forme mère alluaudite est le groupe *C*2/*c* (ou *I*2/*a*). Le choix du sous-groupe d'espace réduit (*P*2_1_/*c*) dans la forme wyllieite provoque un effet sur les positions équivalentes de la structure alluaudite et conduit à un éclatement respectif des deux sites *X*1 e t *M*2 (Moore & Molin-Case, 1974).

### Experimental

La synthèse de la phase Ag_1.09_Mn_3.46_(AsO_4_)_3_ a été réalisée dans un creuset en porcelaine. Les réactifs, AgNO_3_ (Fluka, 85230), C_9_H_9_MnO_6_^.^2H_2_O (Fluka, 63538) e t NH_4_H_2_AsO_4_ (préparé au laboratoire, ASTM 01–775), sont pris dans les proportions Ag:Mn:As égales à 3:5:6. Le mélange finement broyé, est préchauffé lentement dans un four à moufle jusqu'à 623 K en vue d'éliminer NH_3_, H_2_O, CO_2_ et NO_2_. Il est ensuite porté jusqu'à une température de synthèse proche de celle de la fusion à 1143 K. Le produit est alors abandonné à cette température pendant 4 semaines pour favoriser la germination et la croissance des cristaux. Le résidu final a subi en premier lieu un refroidissement lent (5 K/demi journée) jusqu'à 1043 K puis rapide (50 K.h^-1^) jusqu'à la température ambiante. Des cristaux de couleur rouge, de taille suffisante pour les mesures des intensités, ont été séparés du flux par l'eau bouillante. Une analyse qualitative au M.E.B de marque FEI et de type Quanta 200 confirme la présence des différents éléments chimiques attendus: As, Mn, Ag et l'oxygène.

### Refinement

*L*'examen de Fourier-Différence révèle que l'ion Mn3 présente une agitation thermique élevée comparait à celle de Mn1 et Mn2. Dans l'affinement final, et pour des raisons de neutralité électrique, les taux d'occupation des cations Mn3^2+^, Ag1^+^ et Ag2^+^ ont été menés en utilisant la condition SUMP autorisée par le programme *SHELX. L*'affinement de tous les paramètres variables conduit à des ellipsoïdes bien définis. Les densités d'électrons maximum et minimum restants dans la Fourier-différence sont situées respectivements à 0,79 Å de A s2 et à 0,54 Å de Mn4.

### Crystal data

**Table:**

Ag_1.09_Mn_3.46_(AsO_4_)_3_	*F*(000) = 1330
*M**_r_* = 724.02	*D*_x_ = 4.919 Mg m^−^^3^
Monoclinic, *P*2_1_/*c*	Mo *K*α radiation, λ = 0.71073 Å
Hall symbol: -P 2ybc	Cell parameters from 25 reflections
*a* = 6.7470 (7) Å	θ = 11–15°
*b* = 12.9820 (9) Å	µ = 16.64 mm^−^^1^
*c* = 11.2970 (8) Å	*T* = 298 K
β = 98.85 (3)°	Prism, red
*V* = 977.72 (17) Å^3^	0.25 × 0.15 × 0.10 mm
*Z* = 4	

### Data collection

**Table:**

Enraf–Nonius CAD-4 diffractometer	1704 reflections with *I* > 2σ(*I*)
Radiation source: fine-focus sealed tube	*R*_int_ = 0.038
Graphite monochromator	θ_max_ = 27.0°, θ_min_ = 2.4°
ω/2θ scans	*h* = −8→0
Absorption correction: ψ scan (North *et al.*, 1968)	*k* = −1→16
*T*_min_ = 0.071, *T*_max_ = 0.210	*l* = −14→14
2522 measured reflections	2 standard reflections every 120 min
2138 independent reflections	intensity decay: 1.1%

### Refinement

**Table:**

Refinement on *F*^2^	Primary atom site location: structure-invariant direct methods
Least-squares matrix: full	Secondary atom site location: difference Fourier map
*R*[*F*^2^ > 2σ(*F*^2^)] = 0.032	*w* = 1/[σ^2^(*F*_o_^2^) + (0.0433*P*)^2^ + 6.7709*P*] where *P* = (*F*_o_^2^ + 2*F*_c_^2^)/3
*wR*(*F*^2^) = 0.098	(Δ/σ)_max_ < 0.001
*S* = 1.08	Δρ_max_ = 1.08 e Å^−^^3^
2138 reflections	Δρ_min_ = −1.37 e Å^−^^3^
188 parameters	Extinction correction: *SHELXL97* (Sheldrick, 2008), Fc^*^=kFc[1+0.001xFc^2^λ^3^/sin(2θ)]^-1/4^
1 restraint	Extinction coefficient: 0.00067 (14)

### Special details

**Table:**

Geometry. All e.s.d.'s (except the e.s.d. in the dihedral angle between two l.s. planes) are estimated using the full covariance matrix. The cell e.s.d.'s are taken into account individually in the estimation of e.s.d.'s in distances, angles and torsion angles; correlations between e.s.d.'s in cell parameters are only used when they are defined by crystal symmetry. An approximate (isotropic) treatment of cell e.s.d.'s is used for estimating e.s.d.'s involving l.s. planes.
Refinement. Refinement of *F*^2^ against ALL reflections. The weighted *R*-factor *wR* and goodness of fit *S* are based on *F*^2^, conventional *R*-factors *R* are based on *F*, with *F* set to zero for negative *F*^2^. The threshold expression of *F*^2^ > σ(*F*^2^) is used only for calculating *R*-factors(gt) *etc*. and is not relevant to the choice of reflections for refinement. *R*-factors based on *F*^2^ are statistically about twice as large as those based on *F*, and *R*- factors based on ALL data will be even larger.

### Fractional atomic coordinates and isotropic or equivalent isotropic displacement parameters (Å^2^)

**Table:**

	*x*	*y*	*z*	*U*_iso_*/*U*_eq_	Occ. (<1)
As1	0.58985 (10)	0.11831 (5)	0.23334 (6)	0.00808 (18)	
As2	0.87952 (9)	0.39792 (5)	0.26469 (6)	0.00878 (18)	
As3	0.23349 (9)	0.28782 (5)	−0.00773 (6)	0.00740 (18)	
Mn1	0.40485 (14)	0.35350 (7)	0.27517 (9)	0.0080 (2)	
Mn2	0.73285 (15)	0.23422 (8)	0.49855 (8)	0.0099 (2)	
Mn3	0.5000	0.0000	0.5000	0.0143 (5)	0.916 (5)
Mn4	0.07641 (16)	0.16561 (8)	0.21311 (9)	0.0144 (2)	
Ag1	0.75155 (11)	−0.01201 (7)	0.00310 (6)	0.0239 (3)	0.774 (3)
Ag2	0.0000	0.5000	0.0000	0.0305 (5)	0.631 (4)
O1	0.5493 (7)	0.0060 (3)	0.1633 (4)	0.0126 (10)	
O2	0.7681 (7)	0.1779 (3)	0.1698 (4)	0.0132 (9)	
O3	0.6539 (7)	0.1019 (3)	0.3815 (4)	0.0130 (10)	
O4	0.3803 (7)	0.1906 (3)	0.2135 (4)	0.0117 (9)	
O5	0.0876 (7)	0.3269 (4)	0.2693 (4)	0.0172 (10)	
O6	0.2259 (7)	−0.0884 (4)	0.3793 (4)	0.0136 (10)	
O7	0.0586 (8)	0.0122 (4)	0.1655 (4)	0.0191 (11)	
O8	0.7207 (6)	0.3394 (3)	0.3436 (4)	0.0104 (9)	
O9	0.4128 (6)	0.2108 (3)	−0.0446 (4)	0.0092 (9)	
O10	0.1085 (8)	0.1383 (4)	0.3815 (5)	0.0209 (11)	
O11	0.3397 (7)	0.3798 (3)	0.0854 (4)	0.0155 (10)	
O12	0.0524 (7)	0.2180 (4)	0.0434 (4)	0.0137 (10)	

### Atomic displacement parameters (Å^2^)

**Table:**

	*U*^11^	*U*^22^	*U*^33^	*U*^12^	*U*^13^	*U*^23^
As1	0.0147 (3)	0.0049 (3)	0.0058 (3)	−0.0002 (2)	0.0051 (2)	0.0003 (2)
As2	0.0092 (3)	0.0131 (3)	0.0046 (3)	−0.0024 (2)	0.0028 (2)	0.0001 (2)
As3	0.0097 (3)	0.0049 (3)	0.0088 (3)	0.0000 (2)	0.0049 (2)	−0.0016 (2)
Mn1	0.0108 (5)	0.0069 (4)	0.0065 (5)	−0.0005 (3)	0.0021 (4)	−0.0010 (3)
Mn2	0.0142 (5)	0.0077 (5)	0.0072 (5)	−0.0006 (4)	0.0001 (4)	−0.0010 (3)
Mn3	0.0232 (9)	0.0055 (7)	0.0182 (9)	0.0008 (6)	0.0161 (7)	0.0004 (6)
Mn4	0.0176 (5)	0.0147 (5)	0.0114 (5)	−0.0004 (4)	0.0039 (4)	−0.0010 (4)
Ag1	0.0227 (4)	0.0383 (5)	0.0117 (4)	−0.0005 (3)	0.0058 (3)	0.0009 (3)
Ag2	0.0409 (9)	0.0123 (7)	0.0465 (10)	0.0030 (6)	0.0331 (7)	−0.0008 (6)
O1	0.022 (2)	0.006 (2)	0.010 (2)	−0.0022 (18)	0.0046 (19)	−0.0020 (17)
O2	0.021 (2)	0.008 (2)	0.012 (2)	0.0001 (19)	0.0088 (19)	0.0036 (18)
O3	0.027 (3)	0.008 (2)	0.004 (2)	0.0001 (19)	0.0034 (19)	0.0011 (17)
O4	0.018 (2)	0.011 (2)	0.007 (2)	0.0017 (18)	0.0019 (18)	−0.0006 (17)
O5	0.009 (2)	0.032 (3)	0.012 (2)	−0.002 (2)	0.0069 (18)	0.003 (2)
O6	0.021 (2)	0.013 (2)	0.005 (2)	0.0040 (19)	−0.0025 (18)	−0.0011 (18)
O7	0.026 (3)	0.024 (3)	0.008 (2)	0.016 (2)	0.004 (2)	0.005 (2)
O8	0.010 (2)	0.012 (2)	0.012 (2)	0.0018 (17)	0.0083 (18)	0.0055 (18)
O9	0.010 (2)	0.013 (2)	0.006 (2)	0.0021 (17)	0.0054 (17)	−0.0005 (17)
O10	0.033 (3)	0.011 (2)	0.018 (3)	−0.014 (2)	−0.001 (2)	0.003 (2)
O11	0.023 (3)	0.009 (2)	0.015 (2)	−0.0083 (19)	0.005 (2)	−0.0061 (19)
O12	0.013 (2)	0.019 (2)	0.010 (2)	−0.0068 (19)	0.0066 (18)	−0.0042 (19)

### Geometric parameters (Å, º)

**Table:**

As1—O1	1.661 (4)	Mn2—O9^iv^	2.256 (4)
As1—O3	1.677 (4)	Mn2—O6^vi^	2.333 (5)
As1—O2	1.681 (5)	Mn3—O11^iv^	2.204 (5)
As1—O4	1.683 (5)	Mn3—O11^vii^	2.204 (5)
As2—O5^i^	1.674 (5)	Mn3—O3	2.246 (5)
As2—O8	1.677 (4)	Mn3—O3^vi^	2.246 (5)
As2—O6^ii^	1.682 (4)	Mn3—O6^vi^	2.413 (5)
As2—O7^ii^	1.701 (5)	Mn3—O6	2.413 (5)
As3—O9	1.671 (4)	Mn4—O10	1.915 (5)
As3—O11	1.678 (4)	Mn4—O12	2.017 (5)
As3—O12	1.693 (5)	Mn4—O7	2.062 (5)
As3—O10^iii^	1.695 (5)	Mn4—O2^viii^	2.069 (5)
Mn1—O1^ii^	2.105 (4)	Mn4—O4	2.075 (5)
Mn1—O11	2.148 (5)	Mn4—O5	2.186 (6)
Mn1—O5	2.159 (5)	Ag1—O1	2.439 (5)
Mn1—O8	2.160 (5)	Ag1—O7^ix^	2.454 (5)
Mn1—O9^iv^	2.193 (4)	Ag1—O1^ix^	2.547 (5)
Mn1—O4	2.224 (4)	Ag1—O7^i^	2.566 (5)
Mn2—O3	2.183 (5)	Ag2—O10^x^	2.418 (5)
Mn2—O8	2.212 (4)	Ag2—O10^iii^	2.418 (5)
Mn2—O12^v^	2.226 (5)	Ag2—O6^iii^	2.478 (5)
Mn2—O2^iv^	2.227 (5)	Ag2—O6^x^	2.478 (5)
			
O1—As1—O3	111.2 (2)	O3—Mn2—O9^iv^	88.97 (17)
O1—As1—O2	106.1 (2)	O8—Mn2—O9^iv^	73.49 (16)
O3—As1—O2	113.2 (2)	O12^v^—Mn2—O9^iv^	145.34 (18)
O1—As1—O4	110.7 (2)	O2^iv^—Mn2—O9^iv^	89.79 (17)
O3—As1—O4	106.6 (2)	O3—Mn2—O6^vi^	73.47 (17)
O2—As1—O4	109.1 (2)	O8—Mn2—O6^vi^	162.80 (17)
O5^i^—As2—O8	109.6 (2)	O12^v^—Mn2—O6^vi^	93.92 (17)
O5^i^—As2—O6^ii^	108.4 (2)	O2^iv^—Mn2—O6^vi^	85.10 (17)
O8—As2—O6^ii^	110.6 (2)	O9^iv^—Mn2—O6^vi^	114.06 (17)
O5^i^—As2—O7^ii^	108.7 (3)	O11^iv^—Mn3—O11^vii^	180.0 (2)
O8—As2—O7^ii^	106.3 (2)	O11^iv^—Mn3—O3	98.46 (17)
O6^ii^—As2—O7^ii^	113.2 (2)	O11^vii^—Mn3—O3	81.54 (17)
O9—As3—O11	109.1 (2)	O11^iv^—Mn3—O3^vi^	81.54 (17)
O9—As3—O12	110.6 (2)	O11^vii^—Mn3—O3^vi^	98.46 (17)
O11—As3—O12	115.4 (2)	O3—Mn3—O3^vi^	180.0
O9—As3—O10^iii^	116.9 (2)	O11^iv^—Mn3—O6^vi^	78.51 (17)
O11—As3—O10^iii^	100.1 (2)	O11^vii^—Mn3—O6^vi^	101.49 (17)
O12—As3—O10^iii^	104.6 (3)	O3—Mn3—O6^vi^	70.87 (17)
O1^ii^—Mn1—O11	100.20 (18)	O3^vi^—Mn3—O6^vi^	109.13 (17)
O1^ii^—Mn1—O5	104.8 (2)	O11^iv^—Mn3—O6	101.49 (17)
O11—Mn1—O5	86.86 (18)	O11^vii^—Mn3—O6	78.51 (17)
O1^ii^—Mn1—O8	82.81 (18)	O3—Mn3—O6	109.13 (17)
O11—Mn1—O8	114.17 (18)	O3^vi^—Mn3—O6	70.87 (17)
O5—Mn1—O8	156.39 (18)	O6^vi^—Mn3—O6	180.00 (19)
O1^ii^—Mn1—O9^iv^	94.00 (18)	O10—Mn4—O12	170.8 (2)
O11—Mn1—O9^iv^	163.49 (17)	O10—Mn4—O7	94.2 (2)
O5—Mn1—O9^iv^	81.37 (18)	O12—Mn4—O7	94.9 (2)
O8—Mn1—O9^iv^	75.76 (16)	O10—Mn4—O2^viii^	101.8 (2)
O1^ii^—Mn1—O4	175.83 (18)	O12—Mn4—O2^viii^	79.54 (19)
O11—Mn1—O4	81.08 (17)	O7—Mn4—O2^viii^	89.83 (19)
O5—Mn1—O4	79.20 (19)	O10—Mn4—O4	93.8 (2)
O8—Mn1—O4	93.05 (17)	O12—Mn4—O4	83.35 (18)
O9^iv^—Mn1—O4	85.35 (17)	O7—Mn4—O4	99.78 (19)
O3—Mn2—O8	91.75 (17)	O2^viii^—Mn4—O4	161.04 (18)
O3—Mn2—O12^v^	119.67 (18)	O10—Mn4—O5	84.0 (2)
O8—Mn2—O12^v^	85.69 (17)	O12—Mn4—O5	86.98 (19)
O3—Mn2—O2^iv^	155.86 (18)	O7—Mn4—O5	177.62 (19)
O8—Mn2—O2^iv^	110.95 (18)	O2^viii^—Mn4—O5	89.03 (18)
O12^v^—Mn2—O2^iv^	71.89 (18)	O4—Mn4—O5	81.91 (17)

Symmetry codes: (i) *x*+1, *y*, *z*; (ii) −*x*+1, *y*+1/2, −*z*+1/2; (iii) *x*, −*y*+1/2, *z*−1/2; (iv) *x*, −*y*+1/2, *z*+1/2; (v) *x*+1, −*y*+1/2, *z*+1/2; (vi) −*x*+1, −*y*, −*z*+1; (vii) −*x*+1, *y*−1/2, −*z*+1/2; (viii) *x*−1, *y*, *z*; (ix) −*x*+1, −*y*, −*z*; (x) −*x*, *y*+1/2, −*z*+1/2.

## References

[bb1] Alvarez-Vega, M., Gallardo-Amores, J. M., Garcia-Alvarado, F. & Amador, U. (2006). *Solid State Sci.* **8**, 952–957.

[bb2] Antenucci, D., Fransolet, A. M., Miehe, G. & Tarte, P. (1995). *Eur. J. Mineral.* **7**, 175–179.

[bb3] Ayed, B., Abbdallah, A. H. & Hadded, A. (2004). *Acta Cryst.* E**60**, i52–i54.

[bb4] Borel, M. M., Leclaire, A., Chardon, J., Provost, J., Rebbah, H. & Raveau, B. (1997). *J. Solid State Chem.* **132**, 41–46.

[bb5] Brandenburg, K. (1998). *DIAMOND* University of Bonn, Germany.

[bb6] Brown, I. D. & Altermatt, D. (1985). *Acta Cryst.* B**41**, 244–247.

[bb7] Chouaibi, N., Daidouh, A., Pico, C., Santrich, A. & Veiga, M. L. (2001). *J. Solid State Chem.* **159**, 46–50.

[bb8] Duisenberg, A. J. M. (1992). *J. Appl. Cryst.* **25**, 92–96.

[bb9] Farrugia, L. J. (1999). *J. Appl. Cryst.* **32**, 837–838.

[bb10] Fransolet, A.-M. (1995). *Eur. J. Mineral.* **7**, 567–575.

[bb11] Frigui, W., Ben Amor, F., Zid, M. F. & Driss, A. (2011*b*). *Jordan J. Chem.* **6**, 295–305.

[bb12] Frigui, W., Falah, C., Boughzala, H., Zid, M. F. & Driss, A. (2010). *J. Soc. Chim. Tunis*, **12**, 179–188.

[bb13] Frigui, W., Falah, C., Ferhi, M., Zid, M. F. & Driss, A. (2011*a*). *Ann. Chim. Sci. Mat.* **36**, 111–123.

[bb14] Haddad, A., Jouini, T. & Piffard, Y. (1992). *Eur. J. Solid State Inorg. Chem.* **29**, 57–63.

[bb15] Hajji, M. & Zid, M. F. (2006). *Acta Cryst.* E**62**, i114–i116.

[bb16] Harms, K. & Wocadlo, S. (1995). *XCAD4* University of Marburg, Germany.

[bb17] Hatert, F. (2006). *Acta Cryst.* C**62**, i1–i2. 10.1107/S0108270105037236 16397318

[bb18] Korzenski, M. B., Schimek, G. L. & Kolis, J. W. (1998). *J. Solid State Chem.* **106**, 301–309.

[bb19] Leclaire, A., Borel, M. M. & Raveau, B. (2002). *J. Solid State Chem.* **163**, 534–539.

[bb20] Lii, K. H., Tsai, H. J. & Wang, S. L. (1990). *J. Solid State Chem.* **87**, 396–401.

[bb21] Macíček, J. & Yordanov, A. (1992). *J. Appl. Cryst.* **25**, 73–80.

[bb22] MacKay, R., Wardojo, T. A. & Hwu, S.-J. (1996). *J. Solid State Chem.* **125**, 255–260.

[bb23] Masquelier, C., D’Yvoire, F. & Collin, G. (1995). *J. Solid State Chem.* **118**, 33–42.

[bb24] Moore, P. B. & Ito, J. (1973). *Mineral. Rec.* **4**, 131–136.

[bb25] Moore, P. B. & Ito, J. (1979). *Mineral. Mag.* **43**, 227–235.

[bb26] Moore, P. B. & Molin-Case, J. (1974). *Am. Mineral.* **59**, 280–290.

[bb27] North, A. C. T., Phillips, D. C. & Mathews, F. S. (1968). *Acta Cryst.* A**24**, 351–359.

[bb28] Pertlik, F. (1987). *Acta Cryst.* C**43**, 381–383.

[bb29] Sheldrick, G. M. (2008). *Acta Cryst.* A**64**, 112–122. 10.1107/S0108767307043930 18156677

[bb30] Warner, T. E., Milius, W. & Maier, J. (1993). *J. Solid State Chem.* **106**, 301–309.

[bb31] Yakubovich, O. V., Massa, W., Gavrilenko, P. G. & Dimitrova, O. V. (2005). *Eur. J. Mineral.* **17**, 741–747.

[bb32] Zid, M. F., Driss, A. & Jouini, T. (2005). *Acta Cryst.* E**61**, i46–i48.

